# Whole genome sequencing enhances molecular diagnosis of primary ciliary dyskinesia

**DOI:** 10.1002/ppul.27200

**Published:** 2024-08-08

**Authors:** Holly A. Black, Sophie Marion de Proce, Jose L. Campos, Alison Meynert, Mihail Halachev, Joseph A. Marsh, Robert A. Hirst, Chris O'Callaghan, Amelia Shoemark, Daniel Toddie‐Moore, Scottish Genomes Partnership, Javier Santoyo‐Lopez, Jennie Murray, Kenneth Macleod, Don S. Urquhart, Stefan Unger, Timothy J. Aitman, Pleasantine Mill

**Affiliations:** ^1^ Centre for Genomic and Experimental Medicine, MRC Institute of Genetics and Cancer University of Edinburgh Edinburgh UK; ^2^ South East of Scotland Genetics Service Western General Hospital Edinburgh UK; ^3^ MRC Human Genetics Unit, MRC Institute of Genetics and Cancer University of Edinburgh Edinburgh UK; ^4^ Department of Respiratory Sciences, Centre for PCD Diagnosis and Research University of Leicester Leicester UK; ^5^ School of Medicine, Division of Molecular and Clinical Medicine University of Dundee Dundee UK; ^6^ Edinburgh Genomics Edinburgh UK; ^7^ Department of Paediatric Respiratory and Sleep Medicine Royal Hospital for Sick Children Edinburgh UK; ^8^ Department of Child Life and Health University of Edinburgh Edinburgh UK

**Keywords:** ciliopathies, molecular diagnosis, primary ciliary dyskinesia, rare respiratory disease, whole genome sequencing

## Abstract

**Background:**

Primary ciliary dyskinesia (PCD) is a genetic disorder affecting motile cilia. Most cases are inherited recessively, due to variants in >50 genes that result in abnormal or absent motile cilia. This leads to chronic upper and lower airway disease, subfertility, and laterality defects. Given overlapping clinical features and genetic heterogeneity, diagnosis can be difficult and often occurs late. Of those tested an estimated 30% of genetically screened PCD patients still lack a molecular diagnosis. A molecular diagnosis allows for appropriate clinical management including prediction of phenotypic features correlated to genotype. Here, we aimed to identify how readily a genetic diagnosis could be made using whole genome sequencing (WGS) to facilitate identification of pathogenic variants in known genes as well as novel PCD candidate genes.

**Methods:**

WGS was used to screen for pathogenic variants in eight patients with PCD.

**Results:**

7/8 cases had homozygous or biallelic variants in *DNAH5*, *DNAAF4* or *DNAH11* classified as pathogenic or likely pathogenic. Three identified variants were deletions, ranging from 3 to 13 kb, for which WGS identified precise breakpoints, permitting confirmation by Sanger sequencing. WGS yielded identification of a de novo variant in a novel PCD gene *TUBB4B*.

**Conclusion:**

Here, WGS uplifted genetic diagnosis of PCD by identifying structural variants and novel modes of inheritance in new candidate genes. WGS could be an important component of the PCD diagnostic toolkit, increasing molecular diagnostic yield from current (70%) levels, and enhancing our understanding of fundamental biology of motile cilia and variants in the noncoding genome.

## 1 INTRODUCTION

Primary ciliary dyskinesia (PCD, OMIM: PS244400) is a genetic disorder of motile cilia,^1^, ^2^, ^3^ which are highly structurally organized organelles that project from the surface of the cells. Motile cilia beat in a coordinated manner to effectively propel fluids across the surface of the airways, brain ventricles and reproductive tracts. In PCD, these cilia have an abnormal structure and/or function.^2^, ^3^ This can result in a chronic respiratory disease, due to impaired mucociliary clearance, as well as hydrocephalus, laterality defects (e.g., situs inversus) and fertility defects in a subset of patients. PCD often presents at birth as unexplained neonatal respiratory distress. Affected children then develop variable clinical features including a daily wet cough, chronic respiratory tract infections, rhinosinusitis, and otitis media. Over time, these can lead to debilitating long‐term complications, including bronchiectasis and hearing impairment.^1^, ^2^, ^3^, ^4^, ^5^, ^6^

PCD is a clinically heterogeneous disorder that has significant phenotypic overlap with other genetic respiratory diseases, such as cystic fibrosis and primary immunodeficiency. The diagnostic strategy for suspected PCD cases requires a battery of highly specialized tests.^7^, ^8^ This includes detection of low nasal nitric oxide (nNO), as well as high‐speed video microscopy (HSVM) to assess cilia beating pattern and frequency, transmission electron microscopy (TEM) to assess the cilia ultrastructure and immunofluorescence of key proteins from a nasal brush biopsy. None of the tests are fully sensitive or specific and therefore need to be used in combination.^9^, ^10^, ^11^, ^12^, ^13^ Genetic testing is generally performed by gene panel or occasionally by whole exome sequencing, with genetic testing happening towards the end of the diagnostic strategy within Europe.^7^, ^12^ Under the ERS guidelines, a PCD diagnosis is confirmed by a hallmark ultrastructural cilia defect by TEM or by biallelic mutations in a known PCD gene.

PCD is underdiagnosed. PCD has an estimated incidence of 1 in 7500 births, rising to 1 in 2300 in endogamous populations.^14^, ^15^ In North America, it is estimated that only 2000 patients have a confirmed diagnosis of PCD as opposed to the predicted ~25,000– 50,000 these rates would suggest to be affected with PCD.^16^ Similar underdiagnosis is reported across Europe where national registries in Denmark, Switzerland, Cyprus, Norway, and United Kingdom account for <25% predicted patients.^17^, ^18^ A large portion of these missing patients are likely adult patients within primary care or bronchiectasis clinics, as suggested by one study which found 12% of bronchiectasis patients had pathogenic variants in known motile ciliopathy genes.^19^ This underdiagnosis worldwide is thought to be due to lack of awareness, disease heterogeneity, and lack of access to the required specialized diagnostic tests.

PCD is also a genetically heterogeneous disorder. The majority of cases are autosomal recessively inherited, with over 50 genes currently associated with PCD.^1^, ^2^, ^6^, ^20^, ^21^ Mutations in these genes account for approximately 70% of PCD cases screened by gene panel, suggesting additional causal genes exist and/or more complicated structural variants (SVs) are being missed in known genes.^22^ Alternately, rare deep intronic variants may also exist, as have been reported in *CCDC39* and *DNAH11*.^23^, ^24^, ^25^ Genes associated with PCD encode proteins involved in cilia structure, cilia assembly or regulatory complexes. There is often a correlation between the affected gene and the defects in cilia motility or ultrastructure observed at diagnosis.^1^, ^6^, ^7^, ^26^ However, this is not always the case: it is estimated that 9%–20% of PCD cases have normal or inconclusive ultrastructure by TEM.^11^ In these unsolved cases where TEM and/or genotyping are normal, a diagnosis is considered highly likely in the presence of clinical symptoms plus a persistently abnormal ciliary beat pattern which doesn't normalize after culture of cells in vitro. In these cases, the patient can be brought back for further testing as new genetic tests come online.^8^ Therefore optimization of genetic testing in the PCD diagnostic pathway provides an accessible diagnostic test, particularly as next generation sequencing (NGS) is increasingly available.^8^, ^27^, ^28^

In this study, we investigated the utility of whole genome sequencing (WGS) to detect disease‐causing variants in children and young adults with PCD. We selected a non‐endogamous population where PCD was confirmed by nasal brushing to test whether WGS could yield a molecular diagnostic. WGS would provide unbiased genome coverage beyond exons of ~50 genes associated with PCD whilst simultaneously detecting a range of variants, from single nucleotide changes, small insertion‐deletions (indels) as well as complicated SVs, which may be missed by targeted sequencing approaches. Eight patients were recruited, alongside parental samples where available. This led to a genetic diagnosis for all eight cases, three of whom had a pathogenic deletion of 3 kb‐13 kb and one patient had a de novo missense variant p.P259L in a novel candidate gene *TUBB4B*.

## 2 MATERIALS AND METHODS

### 2.1 Patient cohort

**Figure 1:**
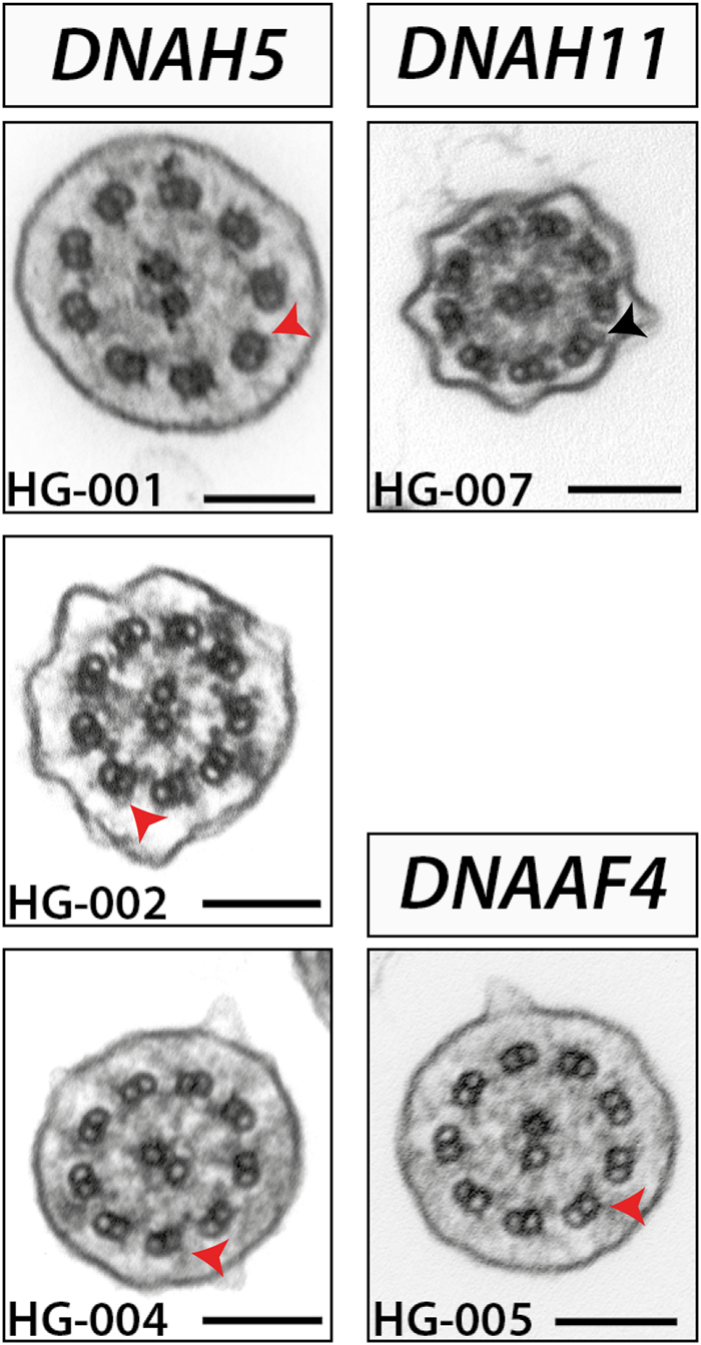
Ultrastructure analysis supports genetic diagnosis for outer arm dynein variants in PCD. (Left) *DNAH5* variants disrupt outer dynein arms (red arrowhead = disrupted, black arrowheads normal) across axonemes of nasal brush samples from cases HG‐001, HG‐002, and HG‐004. (Right) In contrast, *DNAH11* variants do not uniformly disrupt outer dynein arms (black arrowheads) in cilia from nasal brush of case HG‐007. Disruption of both inner and outer dynein arms (red arrowheads) is observed in *DNAAF4* variants, as shown for HG‐005. Scale bars = 100 nm.

All subjects who attended the respiratory clinic at the Royal Hospital for Children and Young People, Edinburgh with a clinical diagnosis of PCD were consecutively approached. Signed and informed consent was obtained from the affected individual as well as relatives through approved protocols. Clinical phenotypes and diagnostic test results are shown in Table 1. For details of blood samples, DNA extraction, and analysis of sample ethnicity see Supporting Information S1: Supplementary Methods and Figure 1. The study was approved by the London‐West London and Gene Therapy Advisory Committee Research Ethics Committee (REC number 11/LO/0883) and also had NHS Lothian R+D approval (2011/RC/B01).

**Table 1:** Clinical details of PCD cases.

Case number	Samples recruited	Sex	Ethnicity	Age at diagnosis	Age at recruitment	Ciliary ultrastructure	Cilia motility	nNO (ppb)	Current FEV1 (% predicted)	NRD	Chronic wet cough	Regular IV antibiotics	Recurrent infections	Rhinosinusitis	Bronchiectasis	Recurrent ear infection	Hearing loss	Situs inversus	Dextrocardia
1	Trio	F	EUR	12	16	Absent outer dynein arms	Static cilia	<5	83	U	Y	N	Y	Y	N	Y	N	N	N
2	Proband only	M	EUR	9	11	Outer dynein arm defect	Static cilia	8.5	90	Y	Y	Y	N	Y	Y	Y	Y	N	N
3	Trio	F	EUR	11	15	Ciliary agenesis	N/A	15.5	47	Y	Y	Y	Y	Y	Y	N	Y	N	N
4	Trio	M	EUR	5	6	Outer dynein arm defect	Static cilia	75	82	Y	Y	N	Y	Y	N	N	Y	N	N
5	Proband and mother	F	EUR	11	31	Complete absence of dynein arm	Static cilia	U	U	N	Y	U	Y	Y	Y	Y	N	N	N
6	Trio	M	EUR	3	21	Unknown	Unknown	8.5	62	U	Y	Y	Y	Y	Y	N	N	N	N
7	Trio	M	SAS	7	9	Normal	Dysmotile	49.5	87	N	N	N	Y	Y	U	N	N	Y	Y
8	Trio	F	EUR	10	11	Normal	Dysmotile	22	75	U	Y	N	Y	Y	N	Y	N	N	N

Abbreviations: EUR, European; F, female; FEV1, forced expiratory volume in 1 s; IV, intravenous; M, male; N, no; nNO, nasal nitric oxide; NRD, neonatal respiratory distress; ppb, parts per billion; SAS, South Asian; U, unknown; Y, yes.

### 2.2 Whole genome sequencing

DNA was sequenced by WGS at Edinburgh Genomics. Libraries were prepared using the Illumina TruSeq PCR‐free protocol and sequenced on the Illumina HiSeq X platform. The average yield per sample was 136 Gb, with mean coverage of 36X (range 33.9–38.3).

### 2.3 Gene panel, data analysis, and variant classification

A virtual gene panel of 146 genes was created, which included the known “green” (diagnostic grade) 34 PCD genes plus 107 suspected ciliopathy genes that may have respiratory features based on the PCD PanelApp panel (v1.14) with five additional genes identified in the literature (*CFAP300*, *DNAH6*, *DNAJB13*, *STK36*, and *TTC25)*.^29^, ^30^, ^31^ Analysis of variants was carried out as described in Supplementary Methods. Additional analyses were carried out across the *FOXJ1* locus for Case 3, in whom no diagnostic variants were identified using the virtual gene panel. A genome‐wide expanded analysis identified a *de novo* missense mutation p.P259L (chr9:g.137242994:C>T (hg38)) in the gene *TUBB4B* only in the patient, and not present in either parent.^32^

### 2.4 Modeling of whole exome sequencing data

A whole exome sequencing (WES)‐like subset of the WGS data was obtained by extracting only the reads mapping to the regions in the TWIST Exome Capture Kit (using samtools v1.6) from the BAM file for each sample. WES CNV calling was performed by ExomeDepth (v1.1.15) as detailed in Supporting Information S1: Supplementary Methods.

### 2.5 Homology modelling of DNAH11 and location of missense variants

A homology model of the C‐terminal region of the DNAH11 motor domain (residues 3348‐4504) was built using PHYRE2.^33^, ^34^ The effects of mutations in the C‐terminal domain (CTD) (residues 4124‐4504) on protein stability were modeled with FoldX.^35^ Sequences of human dynein genes were aligned with MUSCLE^36^ and the sequence alignment was visualized with MView.^37^ Further details are given in the Supporting Information S1: Supplementary Methods.

## 3 RESULTS

Nine patients attending RHCYP clinics were approached to take part. Six consented while three declined due to need for additional blood tests. In addition, two adult PCD patients also agreed to join the study. None had a prior genetic diagnosis. All had low nasal nitric oxide (nNO) and most had abnormal ciliary ultrastructure by transmission electron microscopy (TEM). Eight patients with PCD (50% female), aged 6–31 years, were recruited to the study as six trios, one proband‐mother duo and one singleton (Table 1). All eight cases had a clinical diagnosis of PCD, following a nasal brush biopsy. In all cases, nasal brushings were undertaken for clinical suspicion of PCD. Presentations and phenotype severity were varied and are summarized in Table 1.

Using a virtual PCD panel approach to initially screen the WGS, we confirmed a genetic diagnosis for seven of the eight cases (Table 2). Three cases had biallelic pathogenic and/or likely pathogenic variants in the outer dynein arm heavy chain subunit gene *DNAH5*, which has previously been shown to contribute to the largest proportion of PCD cases among populations of European descent.^1^ Case 1 had a hemizygous nonsense variant (c.5281C>T, p.(Arg1761Ter)), inherited from the mother and previously reported in PCD cases,^38^, ^39^ and an overlapping 13 kb deletion on the other allele, inherited from the father (Supporting Information S1: Figure 3). Case 2 had two nonsense variants in *DNAH5*, c.3949C>T, p.(Gln1317Ter), which was previously reported,^40^ and the c.5281C>T, p.(Arg1761Ter) variant. ddPCR was used to phase the variants in Case 2 (Supporting Information S1: Figure 4). Case 4 had a single base pair deletion, resulting in a frameshift and premature termination codon (PTC) (c.10815del, p.(Pro3606Hisfs)), and a single base pair duplication, resulting in direct creation of a PTC (c.13458dup, p.(Asn4487Ter)), which were shown to be in trans through trio‐based phasing. Both variants have been reported previously in PCD cases.^39^ All three cases with biallelic variants in *DNAH5* were shown to have absence or defects of outer dynein arms on TEM (Figure 1), with Case 1 and Case 2 also shown to have static cilia (Table 1, Video S1). This is consistent with loss of DNAH5, which has been shown to result in outer dynein arm truncation and immotile cilia.^39^ All three share a similar clinical phenotype, with chronic wet cough, sinus problems, and recurrent chest infections. Hearing loss was also noted in cases 2 and 4.

**Table 2:** Genetic variants identified in PCD cases.

Case number	Gene	Transcript	dbSNP ID	Variant(s)	Zygosity	gnomAD allele frequency (v4.0.0)	In silico predictions	ACMG/ACGS classification	ClinVar accession number
1	*DNAH5*	NM_001369.3	N/A	c.5272‐955_6197del p.(?)	Compound heterozygous	Absent^a^	N/A	P (PVS1, PM3, PP4)	SCV001334255
			rs148891849	c.5281C>T, p.(Arg1761Ter)^38^, ^39^, ^40^		6 × 10^−5^	N/A	P (PVS1, PM2, PM3_str, PP4)	SCV001334256
2	*DNAH5*	NM_001369.3	rs1769508571	c.3949C>T, p.(Gln1317Ter)^43^	Compound heterozygous	1.6 × 10^−6^	N/A	P (PVS1, PM2, PM3_str, PP4)	SCV001334257
			rs148891849	c.5281C>T, p.(Arg1761Ter)^38^, ^39^, ^40^		6 × 10^−5^	N/A	P (PVS1, PM2, PM3_str, PP4)	SCV001334256
3	*TUBB4B*	NM_006088.6	N/A	c.776C>T, p.(Pro259Leu)^32^	Heterozygous	Absent	Grantham score 98 REVEL 0.94 (damaging) AlphaMissense 0.998 (LP)	LP (PS2_mod, PS4_sup, PM2, PP3)	SCV002770069.1
4	*DNAH5*	NM_001369.3	rs397515540	c.10815del, p.(Pro3606Hisfs)^39^	Compound heterozygous	4.1 × 10^−4^	N/A	P (PVS1, PM2, PM3_str, PP4)	SCV001334258
			rs775696136	c.13458dup, p.(Asn4487Ter)^39^		1.6 × 10^−4^	N/A	P (PVS1, PM2, PM3_str, PP4)	SCV001334259
5	*DNAAF4*	NM_130810.4	N/A	c.784‐1037_894‐2012del p.(?)^44^	Homozygous	2.5 × 10^−4^ b	N/A	P (PVS1, PM3_str, PP4)	SCV001334260
6	*DNAAF4*	NM_130810.4	rs770136467	c.856G>T, p.(Glu286Ter)	Compound heterozygous	2.6 × 10^−5^	N/A	P (PVS1, PM2, PP4)	SCV001334261
			N/A	c.1048‐149_*1048del p.(?)		Absent^a^	N/A	P (PVS1, PM3, PP4)	SCV001334262
7	*DNAH11*	NM_001277115.2	rs72658835	c.13373C>T, p.(Pro4458Leu)^45^, ^46^, ^47^, ^48^	Homozygous	8.4 × 10^−5^	Grantham score 98 REVEL 0.377 (uncertain) AlphaMissense 0.625 (LP)	LP (PM2, PM3, PP3, PP4)	SCV001334263
8	*DNAH11*	NM_001277115.2	rs1450540788	c.10221_10222del, p.(Cys3409Trpfs)	Compound heterozygous	1.5 × 10^−5^	N/A	P (PVS1, PM2, PP4)	SCV001334264
			N/A	c.13288G>C, p.(Gly4430Arg)^49^		2 × 10^−6^	Grantham score 125 REVEL 0.641 (damaging) AlphaMissense 0.813 (LP)	LP (PM1_sup, PM2, PM3_str, PP3, PP4)	SCV001334265

*Note*: Variants were classified according to the ACMG/ACGS guidelines^41^, ^42^; the criteria met for each variant are shown.

Abbreviations: LP, likely pathogenic; N/A, not applicable; P, pathogenic.

^a^ Absent from gnomAD SV and gnomAD CNV.

^b^ Frequency from gnomAD SV.

Two cases had likely pathogenic variants in the cytoplasmic axonemal dynein assembly factor *DNAAF4* (*DYX1C1*), necessary for assembly and/or stability of the inner and outer dynein arms (Table 2).^44^ Case 5 had a homozygous 3.5 kb deletion encompassing exon 7 of *DNAAF4*, which was also heterozygous in the mother. There was no evidence of uniparental disomy and, given Case 5 is also reported to have an affected sibling, it is likely the father also carries the 3.5 kb deletion, although the father's DNA was unavailable. This variant has previously been reported in PCD.^44^ Ultrastructural analysis was consistent with previous reports for *DNAAF4* mutations, with reports of absent dynein arms^44^ (Figure 1) and immotile cilia shown on HSVM. Case 6 had a maternally‐inherited nonsense variant (c.856G>T, p.(Glu286Ter)) and a paternally‐inherited 3.1 kb deletion encompassing the last two exons of *DNAAF4*, which is predicted to be a loss‐of‐function variant, as the resulting transcript would be subject to nonsense‐mediated decay. Case 6 has bronchiectasis with a recurrent need for antibiotics. He has no ear or hearing problems and situs solitus.

A further two cases had pathogenic or likely pathogenic variants in the outer dynein arm heavy chain subunit *DNAH11*. Case 7 had a homozygous missense variant in *DNAH11* (c.13373C>T, p.(Pro4458Leu)) previously reported in PCD cases,^45^, ^46^ at a low frequency in gnomAD and in silico predictions support pathogenicity (Table 2). There was sufficient evidence to classify the variant as likely pathogenic using ACMG guidelines.^41^, ^42^ Case 8 had a heterozygous 2 bp deletion, resulting in a PTC (c.10221_10222del, p.(Cys3409Trpfs)), which was classified as pathogenic (Table 2). The second variant was a heterozygous missense (c.13288G>C, p.(Gly4430Arg)). This variant is at a low frequency in gnomAD and in silico predictions support pathogenicity, but it has not previously been reported in PCD cases. As the variant is in trans with the c.10221_10222del variant, shown by phasing with parental samples, there is sufficient evidence to classify the variant as likely pathogenic (Table 2). Both Case 7 and Case 8 were shown to have a normal cilia ultrastructure with a dysmotile phenotype (Figure 1, Video S2), consistent with loss of DNAH11, which localizes to the proximal portion of respiratory cilia.^50^ There is however phenotypic variability between Case 7 (situs inversus with dextrocardia and recurrent chest infections) and Case 8 (chronic wet cough and recurrent ear infections, with situs solitus).

**Figure 2:**
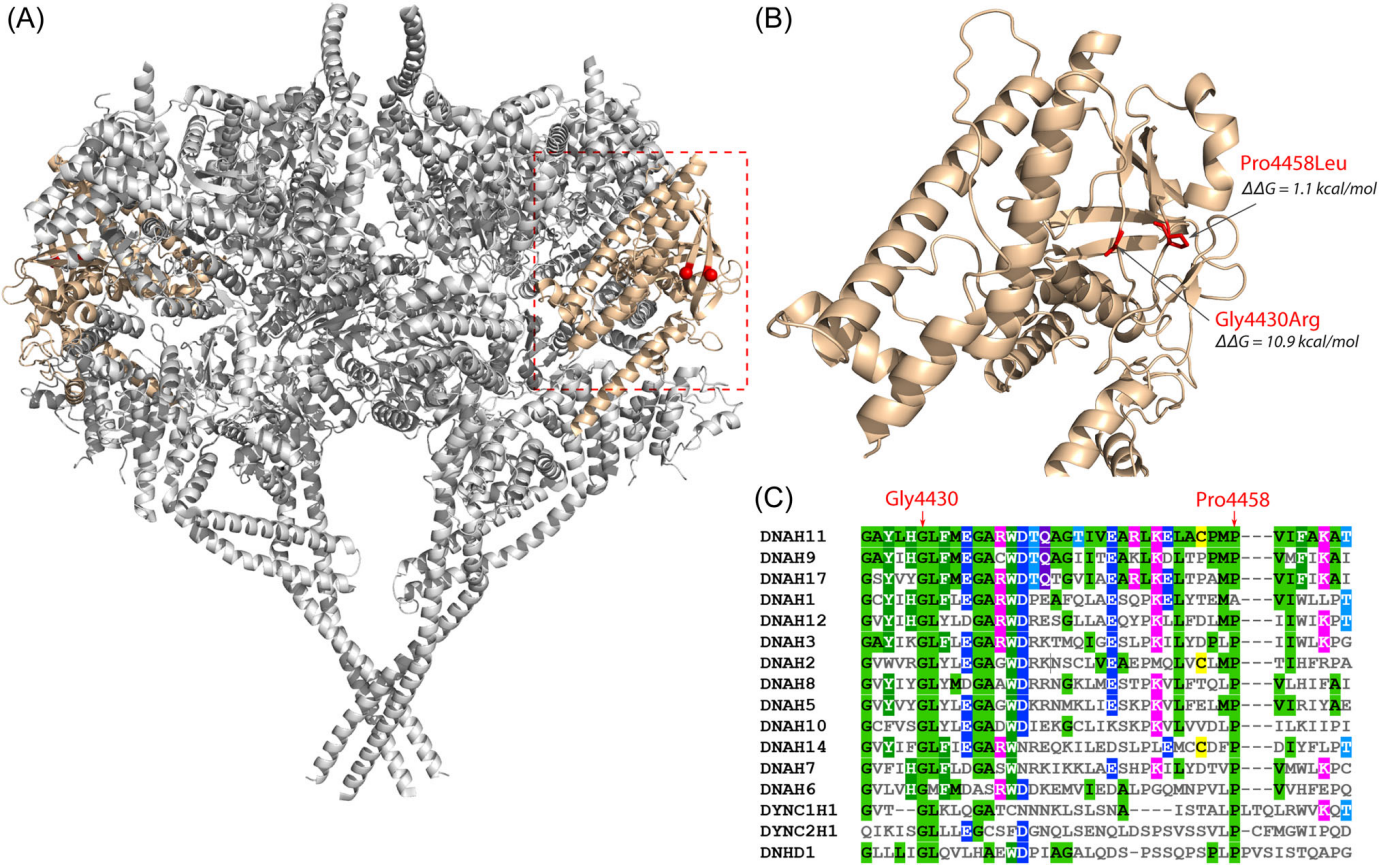
Structural and evolutionary analysis of DNAH11 missense mutations. (A) Structure of the human cytoplasmic dynein‐1 dimer (PDB ID: 5NUG), with the location of the C‐terminal domain colored beige, and the equivalent sites of the DNAH11 mutations highlighted in red. (B) Homology model of the DNAH11 C‐terminal domain with the sites of the missense mutations shown in red, along with the ΔΔG values calculated with FoldX. (C) Multiple sequence alignment of human dynein proteins around the region where the missense mutations occur.

The two missense variants in *DNAH11* both reside at the 3' C‐terminal domain (CTD) of the axonemal dynein heavy chain. While there is no structure available for DNAH11, there are homologous structures of other dynein proteins. To investigate the effects of the *DNAH11* missense mutations on protein structure, we built a homology model of the DNAH11 motor domain. Both mutations occur within the CTD, on the outer side of the dynein motor dimer (Figure 2A) and are intriguingly very close to each other in three‐dimensional space (Figure 2B), separated by only 4.2 Å.

Molecular modelling of the missense mutations using the program FoldX^35^ predicts that the Gly4430Arg should be extremely disruptive to protein structure, with a ΔΔG of 10.9 kcal/mol. We also modeled all other CTD missense variants presented in the gnomAD v2.1 database.^51^ Remarkably, out of 269 variants (Table S1), Gly4430Arg has the highest ΔΔG and therefore is predicted to be the most damaging. Moreover, this position is fully conserved across all human dyneins (Figure 2C). This strongly suggests that Gly4430Arg is pathogenic due to its disruptive effects on protein structure.

In contrast to Gly4430Arg, Pro4458Leu is predicted to be relatively mild at a structural level, with a ΔΔG of 1.1 kcal/mol, making it only the 65th most damaging out of 269 variants (Table S1). However, this residue is highly conserved, existing as a proline across all human dyneins except DNAH1, where it is an alanine. Thus, while Pro4458 is unlikely to cause a severe destablization of protein structure, its remarkable proximity to Gly4430 combined with a moderate structural perturbation and high conservation are supportive of pathogenicity.

To assess whether the three deletion variants identified in Cases 1, 5, and 6 would have been identified using WES, as opposed to WGS, the WGS data was subsetted to create a WES‐like data set for each sample (Supporting Information S1: Figure 2). CNV calling using ExomeDepth was able to identify the *DNAH5* variant c.5272‐955_6197del p.(?) in both the proband and father for Family 1. However, the deletion, which spans five exons and has a breakpoint within exon 37, was called as covering only four of the five affected exons in the proband. The single exon deletion of *DNAAF4* c.784‐1037_894‐2012del p.(?) was identified for the proband in Family 5, where it is homozygous, but not in the heterozygous mother. The *DNAAF4* deletion c.1048‐149_*1048del p.(?) identified in Family 6 was not detected in the proband or father, despite both carrying the variant.

The phenotype of Case 3 is particularly relevant as ultrastructural analysis revealed ciliary agenesis (Video S3), sometimes referred to as reduced generation of multiple motile cilia (RGMC), a specific subtype of PCD. In addition, Case 3 has shunted hydrocephalus, having undergone initial ventriculo‐peritoneal (VP) shunt insertion at 6 weeks, and a subsequent VP shunt revision aged 12 years. To date, few genes have been implicated in this RGMC phenotype by recessive inheritance, *CCNO* (cyclin O) and *MCIDAS* (multicilin)^52^, ^53^ but no pathogenic or potentially pathogenic variants were identified in either gene. Heterozygous de novo mutations in the master motile ciliogenesis transcriptional regulator *FOXJ1* were identified as the first autosomal dominant cause of a distinct PCD‐like condition, associated with chronic respiratory disease, laterality defects and hydrocephalus.^9^ Similar cellular defects are observed with reduced apical docking of centrioles and fewer cilia, but a focused analysis revealed no identifiable pathogenic mutations in the *FOXJ1* locus. An expanded, whole genome analysis for SNP and indel candidates produced a very limited list of variants (Table S2). This included a de novo missense mutation p.P259L (chr9:g.137242994:C>T (hg38)) in the gene *TUBB4B* only in the patient, and not present in either parent or found on gnomAD 4.0 or other publicly available databases. While interpretation of pathogenicity from a single patient is limiting, as part of a large international collaboration, we identified a further 11 patients with PCD carrying heterozygous *TUBB4B* variants identified by next‐generation sequencing (NGS),^32^ indicating it is inherited in an autosomal dominant manner. This included five patients with PCD‐only carrying the identical p.P259L (chr9:g.137242994:C>T) variant. In summary, our WGS strategy was very effective at identifying a novel PCD disease gene with the first report of dominant negative disease mechanisms.

## 4 DISCUSSION

In this study, WGS of affected probands and family members led to a genetic diagnosis in all eight PCD patients, representing 11 different mutations in three autosomal recessive PCD genes and one de novo mutation in a novel autosomal dominant PCD candidate *TUBB4B*. In Case 3, a patient with features of reduced generation of motile cilia and hydrocephalus, no pathogenic variants would been identified in a panel‐based approach, even with targeted sequencing of potential candidates for known RGMC loci. The diagnostic rate of 100% in our small study is higher than previous reports, in which 60%–70% of cases received a genetic diagnosis based on the known PCD gene panels,^1^, ^5^, ^21^, ^22^, ^54^ 75% on extended NGS panels^55^ and 68%–94% by WES.^47^, ^56^, ^57^, ^58^

Next‐generation sequencing (NGS) technologies continue to revolutionize rare genetics research and clinical diagnostics, where the advantages of WES versus WGS are often fiercely debated. Cheaper in terms of costs of sequencing, analysis and data storage, WES is generally preferred as a front‐line diagnostic tool. WES, however, has several issues in terms of evenness of genome coverage and sequence bias, particular for copy number variants (CNV). In comparison, several studies have found more accurate variant calls, as well as even and unbiased coverage of coding regions, are generated by WGS.^24^, ^59^, ^60^ Our analysis was focused on known and candidate PCD genes, screening simultaneously for single nucleotide variants (SNVs), small indels and more complex SVs (Table S3). In three cases, pathogenic deletions were identified, ranging in size from 3 to 13 kb. Modelling of our WGS data to represent WES‐like data suggested that WES data would only have identified the deletion in one of these three cases, in which the deletion was homozygous. Since our WES‐like model has more uniform coverage than true WES data, our WES‐like model could be considered more reliable for CNV calling than real‐life WES sequence. However, we acknowledge that our model is based on 36X WGS data, whereas WES would be nearer 100X in practice but coverage does vary across commercial platforms. The ability to detect SVs in PCD is of importance and consistent with recent diagnostic guidelines for PCD, which highlighted that causal SVs and intronic mutations can be missed due to the large number and size of PCD genes.^8^, ^27^ Further, WES would not have provided the precise breakpoint information that allowed confirmation of the three deletion variants by Sanger sequencing.

Where WGS was clearly advantageous was PCD disease gene discovery in patients without biallelic variants in known genes or in the one recent example of autosomal dominant inheritance (*FOXJ1*) or in the few cases of X‐linked recessive inheritance (*RPGR, PIH1D3, OFD1)*.^21^ In Case 3, WES would have likely identified the *de novo* SNV in *TUBB4B*, as WES panels identified SNVs in the other 11 *TUBB4B* patients.^32^ Here, the clear advantage of WGS was to rule out potential noncoding alterations in the known RGMC PCD genes of known inheritance such as *FOXJ1* as to quickly prioritize novel candidates in our first proband. In our study, WGS was applied to patients with PCD, all having low nNO and most having abnormal ciliary ultrastructure, and where the majority of variants identified were in genes involved axonemal dynein assembly. However, the *TUBB4B* case demonstrates that expanding the application of WGS to a broader cohort of patients with PCD, such as those with normal TEM or nonclassical PCD features, may reveal more fundamental insights into motile cilia and show an even greater diagnostic yield than the current 70% ceiling.^22^ A clear benefit of WGS, similar to WES, is that its findings are future‐proof; the data generated can be re‐screened for PCD‐causing variants identified subsequent to genetic testing, if initial testing is inconclusive. However, only WGS will allow future analysis of noncoding genome for variations in regulatory elements such as transcription factor binding sites that may underlie a subset of unsolved PCD cases.

Whatever the modality, increased genetic testing for PCD is critical. Importantly, a delayed PCD diagnosis is associated with a poorer prognosis.^61^, ^62^ Therefore securing a molecular diagnosis in the neonatal period, by screening term neonates with respiratory distress and/or situs defects by WGS may improve patient outcomes long term.^63^, ^64^ Increasing availability, reducing costs and recognized clinical utility of NGS platforms such as WGS in the neonatal and paediatric intensive care unit setting would be consistent with such indications.^48^, ^60^, ^63^, ^65^, ^66^, ^67^, ^68^ Moreover, either WES or WGS would help rule out other confounding clinical presentations such as primary immune deficiency disorders (PIDs), with overlapping symptoms of frequent, often severe, airway infections as well as recurrent otitis media, and sinusitis.^48^, ^69^, ^70^ Both platforms are advantageous as they allow analysis of all potential causative genes, known and novel, thus faster to keep up with recently reported genes that may not yet be included on PCD‐gene panels. Indeed such technologies may prove to be more cost‐ and time‐effective as providing a molecular diagnosis test for PCD than conventional clinical gene panels.^56^, ^57^, ^58^, ^71^

PCD needs to enter the precision medicine era. A genetic diagnosis is key to improved patient prognosis as we better understand genotype‐phenotype relations^72^ and critically to being trial‐ready as much‐needed genetic therapies come online.^73^, ^74^ Increased genetic testing is also being combined with a curated worldwide database, similar to that of CFTR2 for cystic fibrosis,^75^ to enable a better understanding of these genotype‐phenotype relations in PCD called CiliaVar. Such a database will significantly facilitate interpretation of variants, of which 21% has been suggested to be variants of unknown significance (VUS).^76^ For example, in Cases 7 and 8 in our study, structural modelling of the CTD of *DNAH11* aided the assignment of pathogenicity of two missense variants, with one predicted to be detrimental to protein stability and the second, in close proximity to the first, shown to be highly conserved.

In conclusion, this study demonstrates the benefits of using WGS to obtain a genetic diagnosis for PCD, through its ability to detect SNVs and SVs simultaneously as well as detecting variants in genes outwith current gene panels. The detection of multikilobase deletions in three of the seven diagnosed cases highlights the need to detect SVs as part of the genetic testing for PCD. It also allowed rapid prioritization of SNVs in novel candidate genes with dominant modes of inheritance. Practically and financially, WGS would likely sit behind current standard of care panel‐based or WES diagnostic platforms. Given the high genetic diagnostic rate observed here and elsewhere,^47^, ^55^, ^56^, ^58^ we suggest that genetic testing could be an early step in the current diagnostic pathway for PCD, particularly in cases where nasal brush biopsy is unavailable. A prospective study would appear to be warranted to assess the utility of WGS for PCD in the neonatal period where there is potential for transformative long‐term reduction in morbidity with access to specialist care and disease‐modifying therapies commenced before permanent lung damage.

## AUTHOR CONTRIBUTIONS

**Holly A. Black**: Investigation; validation; formal analysis; data curation; writing—original draft; writing—review & editing. **Sophie Marion de Proce**: Investigation; formal analysis; writing—review & editing. **Jose L Campos**: Writing—review & editing; formal analysis. **Alison Meynert**: Writing—review & editing; formal analysis; data curation; supervision. **Mihail Halachev**: Formal analysis; data curation; writing—review & editing. **Joseph A Marsh**: Formal analysis; visualization; writing—review & editing; methodology. **Robert A Hirst**: Formal analysis; writing—review & editing. **Chris O'Callaghan**: Writing—review & editing; resources. **Amelia Shoemark**: Writing—review & editing; investigation. **Daniel Toddie‐Moore**: Writing—review & editing. **Javier Santoyo‐Lopez**: Writing—review & editing; investigation. **Jennie Murray**: Resources; writing—review & editing. **Kenneth Macleod**: Writing—review & editing; resources. **Don S Urquhart**: Resources; writing—review & editing; project administration; conceptualization. **Stefan Unger**: Writing—review & editing; resources; project administration; conceptualization. **Timothy J Aitman**: Conceptualization; writing—review & editing; project administration; resources; funding acquisition; writing—original draft. **Pleasantine Mill**: Writing—original draft; writing—review & editing; conceptualization; funding acquisition; resources; project administration.

## CONFLICTS OF INTEREST STATEMENT

A. Meynert received core funding from the UKRI Medical Research Council. M. Halachev received core funding from the UKRI Medical Research Council. J. A. Marsh received funding from the UKRI Medical Research Council and Lister Institute. A. Shoemark received consulting fees from Spirovant, Translate Bio and ReCode Therapeutics, as well as payment for events from Ethris, Translate Bio and Insmed. She declares participation in the CARE‐UK Trial steering committee, as well as roles in the European Respiratory Society Clinical Research Collaborations (EMBARC, BEATPCD, AMR), where EMBARC3 is supported by project partners Armata, AstraZeneca, Boehringer Ingelheim, Chiesi, CSL Behring, Grifols, Insmed, Janssen, LifeArc, Novartis, and Zambon. The Scottish Genome Partnership received funding from the UKRI Medical Research Council and the Chief Scientist Office of the Scottish Government. Don Urquhart received funding from the NHS Research Scotland. He declares payment from Vertex Pharmaceuticals for events and participation in AbbVie Pharmaceutical's advisory board. He declares unpaid roles as the deputy chair of the European Cystic Fibrosis Society Exercise Working Group. S. Unger received funding from the NHS Research Scotland. T. J. Aitman acknowledges funding from the UKRI Medical Research Council and the the Chief Scientist Office of the Scottish Government, as co‐investigator for SGP. He declares support and consulting fees from BioCaptiva plc, for which he was former Director and now consultant, as well as shareholder with BioCaptiva plc. P. Mill received funding from the UKRI Medical Research Council and from the European Research Council. She declares payment for an event from Pfizer. She declares her unpaid roles as a member of the Ciliopathy Alliance Scientific Advisory Board, PCD Research Scientific Advisory Panel, the UK Cilia Network leadership team and University of Porto UMIB Scientific Advisory Panel. The remaining authors declare no conflict of interest.

## Supporting information

Supporting information.

Supporting information.

Supporting information.

Supporting information.

Supporting information.

Supporting information.

Supporting information.

Supporting information.

Supporting information.

## Data Availability

The data that support the findings of this study are available from the corresponding author upon reasonable request.
