# Supplementary material for: Whole genome sequencing enhances molecular diagnosis of primary ciliary dyskinesia
Source: Pediatr Pulmonol. 2024 Aug 8;59(12):3322–32. doi: 10.1002/ppul.27200 (PMC11600997; doi:10.1002/ppul.27200)
Supplement: Supplementary file 1 — Supporting information. [file PPUL-59-3322-s003.docx]

**High diagnostic rate of whole genome sequencing in primary ciliary dyskinesia**

**Supplementary Material and Methods**

Holly A Black^1,2^, Sophie Marion de Proce^1*^, Jose L Campos^3*^, Alison Meynert^3^, Mihail Halachev^3^, Joseph A Marsh^3^, Robert A Hirst^4^, Chris O’Callaghan^4^, Scottish Genomes Partnership, Javier Santoyo-Lopez^5^, Jennie Murray^2,3^, Kenneth Macleod^6,7^, Don S Urquhart^6,7^, Stefan Unger^6,7^^, Timothy J Aitman^1^^, Pleasantine Mill^3^^

^1^ Centre for Genomic and Experimental Medicine, MRC Institute of Genetics and Molecular Medicine, University of Edinburgh, Edinburgh, UK

^2^ South East of Scotland Genetics Service, Western General Hospital, Edinburgh, UK

^3^ MRC Human Genetics Unit, MRC Institute of Genetics and Molecular Medicine, University of Edinburgh, Edinburgh, UK

^4^ Centre for PCD Diagnosis and Research, Department of Respiratory Sciences, University of Leicester, UK

^5^ Edinburgh Genomics, Edinburgh, UK

^6^ Department of Paediatric Respiratory and Sleep Medicine, Royal Hospital for Sick Children, Edinburgh, UK

^7^ Department of Child Life and Health, University of Edinburgh, Edinburgh, UK

* These authors contributed equally to the manuscript

^^^ Joint senior authors

**SUPPLEMENTARY MATERIALS**

**Supplementary Table 1: Ranked list of most disruptive reported variants (ΔΔG) in the C-terminal domain (CTD) of DNAH11 as predicted by FoldX.** ΔΔG represents the change in free energy by mutation/design of proteins as predicted by the FoldX algorithm, where ΔΔG = ΔGfold(mutation) − ΔGfold(wild type).

**Supplementary Table 2: Genome-wide list of variants detected in patient HG-003 by Slivar.**

**Supplementary Table 3: Summary of analysis approach used on WGS dataset for each case, first by targeted panel then whole genome level analysis, to identify pathogenic variants.**

**Supplementary File 1: Gene panel used for variant filtering with G2P under a monoallelic inheritance model.**

**Supplementary File 2: Droplet digital PCR for variant phasing.**

**Supplementary Video 1: HSVM for cases 1, 2 and 4, which have a genetic diagnosis in *DNAH5*, showing static cilia**

**Supplementary Video 2: HSVM for cases 7 and 8, which have a genetic diagnosis in *DNAH11*, showing dysmotile cilia**

**Supplementary Video 3: HSVM for case 3, showing ciliary agenesis**

**
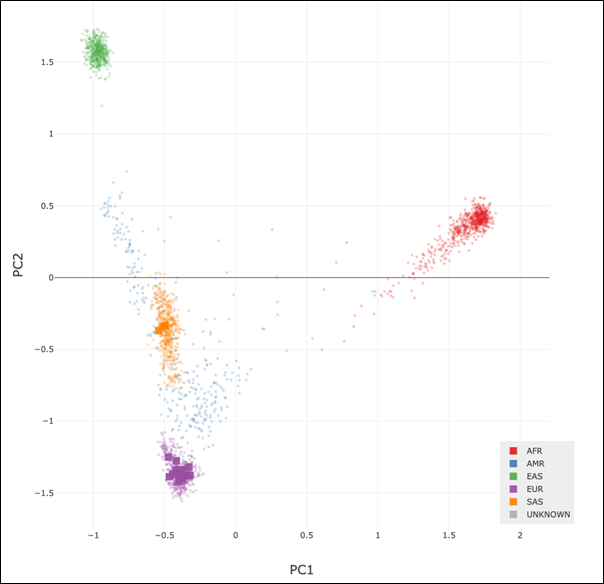
**

**Supplementary Figure 1: Principal component analysis (PCA) of study samples compared to 1000 Genomes project samples.** Peddy was used to predict ancestry of the samples used in the study by comparison with the 1000 Genomes samples. The 1000 Genomes samples (dots) are colour-coded by location. The samples in our study are represented by squares. All samples were predicted to be of European ancestry (purple squares), except three (orange squares), which were predicted to have South Asian ancestry. AFR= African; AMR= American; EAS= East Asian; EUR= European; SAS= South Asian.

**
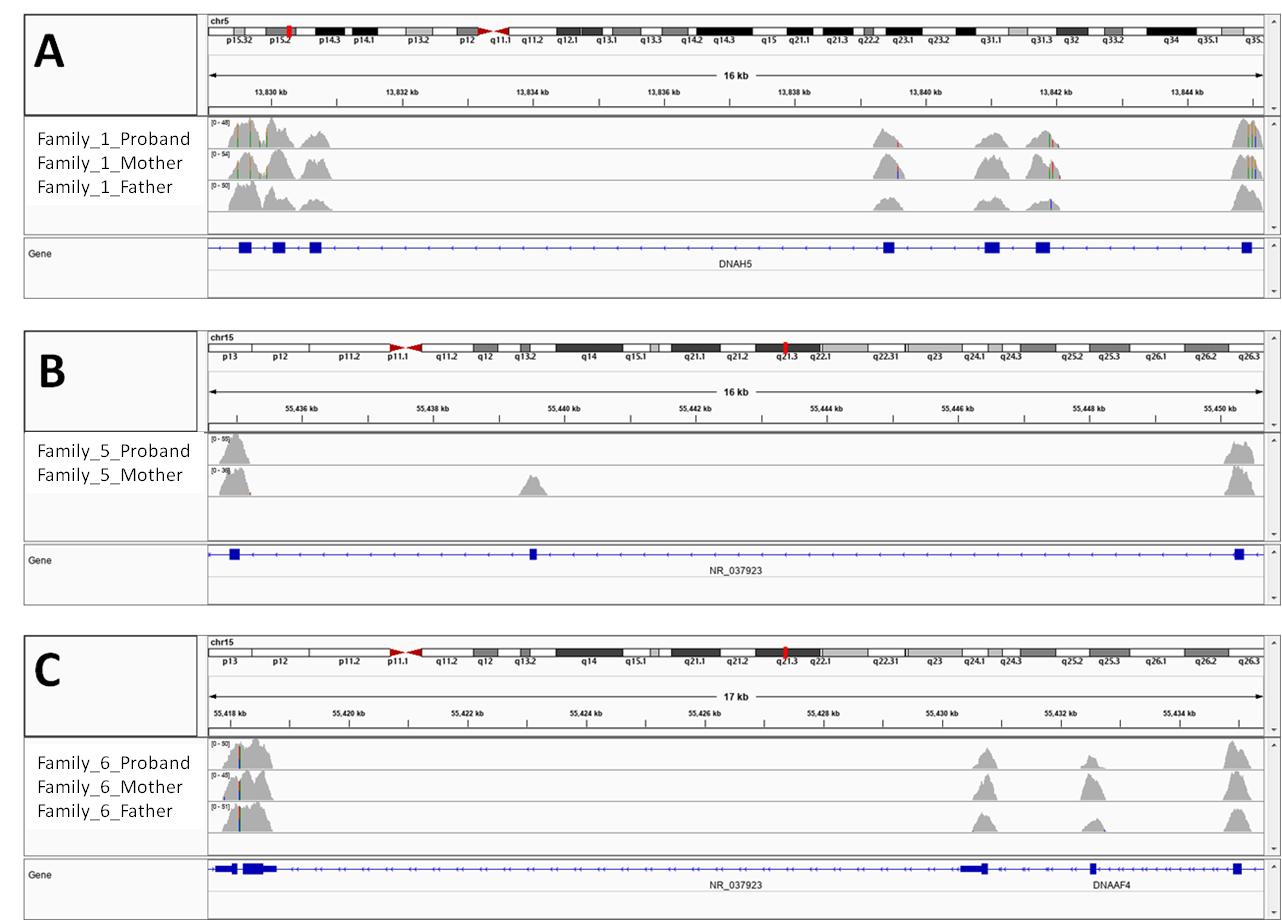
**

**Supplementary Figure 2: Alignments of the modelled WES data showing sufficient coverage to call the deletion variants identified in Cases 1, 5 and 6.** A: Alignment for Family 1, showing coverage of exons 32-38 of *DNAH5.* B: Alignment for Family 5, showing coverage of exons 6 to 8 of *DNAAF4.* C: Alignment for Family 6, showing coverage exons 8-10 of *DNAAF4*.

**
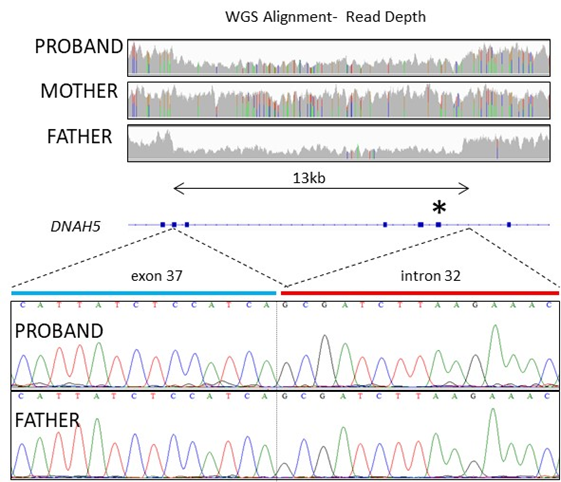
**

**Supplementary Figure 3: Sanger sequencing confirms 13kb deletion in *DNAH5* in Case 1.** Alignments of the WGS data for Family 1 show a drop in read depth to approximately 50% of that of the surrounding regions across a 13kb region of *DNAH5* in the proband and the father. This spans from intron 32 to exon 37. PCR and Sanger sequencing across the breakpoints confirms this deletion. * indicates the position of the c.5281C>T nonsense variant, which is on the maternal haplotype and is therefore hemizygous in the proband.

**
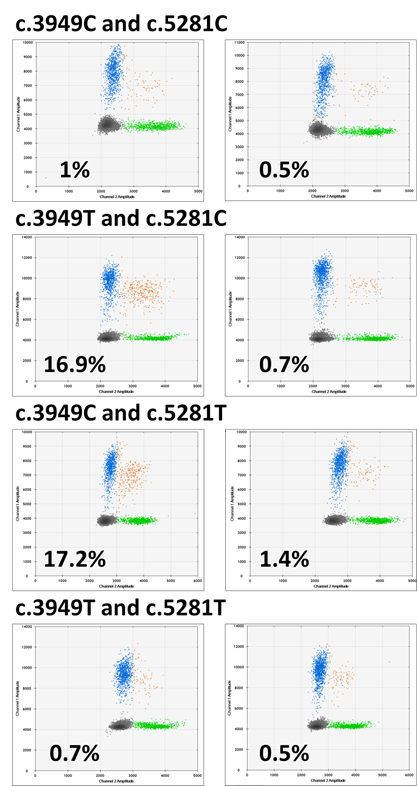
Supplementary Figure 4: Drop phase results confirms the two *DNAH5* nonsense variants are on different haplotypes for Case 2**. The c.3949 variant was assayed using FAM probes and the c.5281 variant was assayed using HEX probes. For each combination of alleles, a representative result from genomic DNA (left) and *Pac*I-digested DNA (right) is shown. Each figure plots the number of FAM-only positive (blue), HEX-only positive (green), FAM and HEX-positive (orange) and negative (grey) droplets. The linkage % is shown for each test. (Details see Supplementary File 2).

**SUPPLEMENTARY METHODS**

*Patient cohort*

Eight young people with PCD (50% female), aged 6 to 31 years (mean=15, SD= 7.9), were recruited to the study within the Department of Paediatric Respiratory and Sleep Medicine at the Royal Hospital for Sick Children, Edinburgh, and the South East Scotland Genetics service. All eight cases had a confirmed clinical diagnosis of PCD, following TEM and/or HSVM of a nasal brush biopsy sample. The screening and diagnostic testing was performed according to the PCD National Service protocols, with investigations including nNO, nasal brush biopsies analysed by HSVM for ciliary beat frequency and pattern and quantitative electron microscopy for ciliary ultrastructure. Clinical phenotypes are shown in **Table 1**. Blood samples were collected in EDTA tubes from the patients and parents, where available. DNA was extracted using the Chemagic DNA blood kit (Chemagen) or the Nucleon Bacc3 kit (GE Healthcare). Sample ethnicity was assessed using Peddy (v4.0.6) [1] (**Supplementary Figure 1**).

*Gene Panel, sequence data analysis and variant classification*

BCBio-Nextgen (0.9.7) was used for alignment and variant detection. This used bwa mem (0.7.13) to align reads to the hg38/GRCh38 reference genome [2], samblaster (0.1.22) to mark duplicate fragments [3] and GATK (3.4-0-g7e26428) for indel realignment and base recalibration [4]. GATK HaplotypeCaller was used to calculate genotype likelihoods. Joint genotyping and quality control, including kinship estimates to confirm sample relatedness, were performed using in-house pipelines with GATK (4.0.2.1) following the GATK best practices. Variants were annotated using Ensembl variant effect predictor (VEP 90) [5].

A bespoke gene panel of 146 genes was created (**Supplementary Files 1**), based on the PCD PanelApp panel (v1.14) and five additional genes identified in the literature (*CFAP300*, *DNAH6*, *DNAJB13*, *STK36* and *TTC25*) [6-8]. Variants (SNPs and small indels) were filtered to retain only those within genes on the gene panel and then further filtered to identify candidate variants by inheritance model (both biallelic and monoallelic), transcript consequence, and population allele frequency using the G2P plugin for VEP [9]. Variants were assessed using Alamut (v2.13) [10] and classified using the ACMG variant interpretation guidelines [11, 12]. Any variants classified as pathogenic or likely pathogenic were validated using Sanger sequencing and were submitted to ClinVar. Parental samples were used to determine the phase of compound heterozygous variants, except for Case 2, which used droplet digital PCR (ddPCR) for phasing (**Supplementary File 2**). A workflow of how the whole genome sequencing dataset was analyzed for each case to make a molecular diagnosis is summarized in **Supplementary Table 3**.

SVs were called using Manta [13] and Canvas (version 1.38) [14]. The detection of SVs can be challenging and it is common practise to use complementary approaches to detect them [15]. Manta detects SVs using discordant paired-end and split reads, whereas Canvas relies on changes in read coverage. We searched for any SVs present in our gene panel that were inherited from either parent. We also searched for de novo SVs for cases where both parents were available. SVs were confirmed *in silico* using SV-Plaudit [16], a tool for rapidly curating SV predictions, and/or using the Integrative Genomics Viewer tool [17]. Candidate variants were confirmed in the laboratory by PCR and Sanger sequencing across the deletion breakpoints.

For Case 3, we did not find any diagnostic variants using our gene panel. As *FOXJ1* was only recently identified as a PCD gene and hence was not present on the panel, we searched for SNVs, indels and SVs in Case 3 in this gene, given the ciliary agenesis phenotype observed in this case is associated with *FOXJ1*. We also expanded our analysis for this case to a genome wide search for SNV and small indel candidates with Slivar (0.1.10), following the protocol for rare diseases (<https://github.com/brentp/slivar/wiki/rare-disease> Date accessed: January 2020). A small number of variants was detected (**Supplementary Table 2**) and none were identified that fitted the current modes of inheritance for PCD.

*Modelling of whole exome sequencing data*

A whole exome sequencing (WES)-like subset of the WGS data was obtained by extracting only the reads mapping to the regions in the TWIST Exome Capture Kit (using samtools v1.6) from the BAM file for each sample. The capture region fully covers the exons affected by copy number variants (CNVs) identified based on the WGS data (**Supplementary Figure 2**). The WES CNV calling was performed using ExomeDepth (v 1.1.15) separately on each individual from the three families in which a pathogenic CNV was identified (Families 1, 5 and 6). As controls, we used WES-like subset data from the WGS data for the other samples in this project (total of 21), from which we excluded any members of the family currently being evaluated. As the WGS data was aligned to GRCh38, we generated a custom reference dataset (exons.GRCh38) required by ExomeDepth, to replace the dataset currently distributed with the ExomeDepth package (exons.hg19); exons.GRCh38  is based on the latest CCDS release (r 22) available for the GRCh38 human genome reference (Available at: [www.ncbi.nlm.nih.gov/projects/CCDS/CcdsBrowse.cgi?REQUEST=SHOW_STATISTICS](http://www.ncbi.nlm.nih.gov/projects/CCDS/CcdsBrowse.cgi?REQUEST=SHOW_STATISTICS) ).

*Homology modelling of DNAH11 and location of missense variants*

A homology model of the C-terminal region of the DNAH11 motor domain (residues 3348-4504) was built using PHYRE2 [18], based upon the cryo-electron microscopy structure of human cytoplasmic dynein-1 (PDB ID: 5NUG) [19]. The effects of the mutations in the C-terminal domain (CTD) (residues 4124-4504) on protein stability were modelled with FoldX [20], using default parameters and calculated over 10 replicates. Sequences of human dynein genes were aligned with MUSCLE [21] and the sequence alignment was visualised with MView [22].

**SUPPLEMENTARY REFERENCES**

1. Pedersen, B.S. and A.R. Quinlan, *Who's Who? Detecting and Resolving Sample Anomalies in Human DNA Sequencing Studies with Peddy.* Am J Hum Genet, 2017. **100**(3): p. 406-413.

2. Li, H. and R. Durbin, *Fast and accurate short read alignment with Burrows-Wheeler transform.* Bioinformatics, 2009. **25**(14): p. 1754-60.

3. Faust, G.G. and I.M. Hall, *SAMBLASTER: fast duplicate marking and structural variant read extraction.* Bioinformatics, 2014. **30**(17): p. 2503-5.

4. McKenna, A., et al., *The Genome Analysis Toolkit: a MapReduce framework for analyzing next-generation DNA sequencing data.* Genome Res, 2010. **20**(9): p. 1297-303.

5. McLaren, W., et al., *The Ensembl Variant Effect Predictor.* Genome Biol, 2016. **17**(1): p. 122.

6. Martin, A.R., et al., *PanelApp crowdsources expert knowledge to establish consensus diagnostic gene panels.* Nat Genet, 2019. **51**(11): p. 1560-1565.

7. Genomics England. *PanelApp Primary ciliary disorders*. Available from: <https://panelapp.genomicsengland.co.uk/panels/178/>.

8. McKusick-Nathans Institute of Genetic Medicine Johns Hopkins University. *Online Mendelian Inheritance in Man, OMIM®*. 11/10/2018].

9. Thormann, A., et al., *Flexible and scalable diagnostic filtering of genomic variants using G2P with Ensembl VEP.* Nat Commun, 2019. **10**(1): p. 2373.

10. SOPHiA GENETICS, *alamut VISUAL*. 2019.

11. Richards, S., et al., *Standards and guidelines for the interpretation of sequence variants: a joint consensus recommendation of the American College of Medical Genetics and Genomics and the Association for Molecular Pathology.* Genet Med, 2015. **17**(5): p. 405-24.

12. Ellard, S., et al. *ACGS Best Practise Guidelines for Variant Classification in Rare Disease 2020*. 2020. 1-33.

13. Chen, X., et al., *Manta: rapid detection of structural variants and indels for germline and cancer sequencing applications.* Bioinformatics, 2016. **32**(8): p. 1220-2.

14. Ivakhno, S., et al., *Canvas SPW: calling de novo copy number variants in pedigrees.* Bioinformatics, 2018. **34**(3): p. 516-518.

15. Cameron, D.L., L. Di Stefano, and A.T. Papenfuss, *Comprehensive evaluation and characterisation of short read general-purpose structural variant calling software.* Nat Commun, 2019. **10**(1): p. 3240.

16. Belyeu, J.R., et al., *SV-plaudit: A cloud-based framework for manually curating thousands of structural variants.* Gigascience, 2018. **7**(7).

17. Robinson, J.T., et al., *Integrative genomics viewer.* Nat Biotechnol, 2011. **29**(1): p. 24-6.

18. Kelley, L.A., et al., *The Phyre2 web portal for protein modeling, prediction and analysis.* Nat Protoc, 2015. **10**(6): p. 845-58.

19. Zhang, K., et al., *Cryo-EM Reveals How Human Cytoplasmic Dynein Is Auto-inhibited and Activated.* Cell, 2017. **169**(7): p. 1303-1314 e18.

20. Guerois, R., J.E. Nielsen, and L. Serrano, *Predicting changes in the stability of proteins and protein complexes: a study of more than 1000 mutations.* J Mol Biol, 2002. **320**(2): p. 369-87.

21. Edgar, R.C., *MUSCLE: multiple sequence alignment with high accuracy and high throughput.* Nucleic Acids Res, 2004. **32**(5): p. 1792-7.

22. Brown, N.P., C. Leroy, and C. Sander, *MView: a web-compatible database search or multiple alignment viewer.* Bioinformatics, 1998. **14**(4): p. 380-1.
